# Arteriovenous metabolomics in pigs reveals *CFTR* regulation of metabolism in multiple organs

**DOI:** 10.1172/JCI174500

**Published:** 2024-05-14

**Authors:** Hosung Bae, Bo Ram Kim, Sunhee Jung, Johnny Le, Dana van der Heide, Wenjie Yu, Sang Hee Park, Brieanna M. Hilkin, Nicholas D. Gansemer, Linda S. Powers, Taekyung Kang, David K. Meyerholz, Victor L. Schuster, Cholsoon Jang, Michael J. Welsh

**Affiliations:** 1 Department of Biological Chemistry, University of California – Irvine, Irvine, California, USA.; 2 Department of Internal Medicine, Pappajohn Biomedical Institute, Roy J. and Lucille A. Carver College of Medicine, University of Iowa, Iowa City, Iowa, USA.; 3 Howard Hughes Medical Institute, University of Iowa, Iowa City, Iowa, USA.; 4 Department of Surgery and; 5 Department of Pathology, Roy J. and Lucille A. Carver College of Medicine, University of Iowa, Iowa City, Iowa, USA.; 6 Department of Internal Medicine, Albert Einstein College of Medicine, Bronx, New York, New York, USA.; 7 Center for Complex Biological Systems and; 8 Center for Epigenetics and Metabolism, University of California – Irvine, Irvine, California, USA.; 9 Department of Molecular Physiology and Biophysics, Pappajohn Biomedical Institute, Roy J. and Lucille A. Carver College of Medicine University of Iowa, Iowa City, Iowa, USA.

**Keywords:** Metabolism, Amino acid metabolism, Ion channels, Monogenic diseases

## Abstract

Mutations in the cystic fibrosis transmembrane conductance regulator (*CFTR*) gene cause cystic fibrosis (CF), a multiorgan disease that is characterized by diverse metabolic defects. However, other than specific *CFTR* mutations, the factors that influence disease progression and severity remain poorly understood. Aberrant metabolite levels have been reported, but whether *CFTR* loss itself or secondary abnormalities (infection, inflammation, malnutrition, and various treatments) drive metabolic defects is uncertain. Here, we implemented comprehensive arteriovenous metabolomics in newborn CF pigs, and the results revealed *CFTR* as a bona fide regulator of metabolism. *CFTR* loss impaired metabolite exchange across organs, including disruption of lung uptake of fatty acids, yet enhancement of uptake of arachidonic acid, a precursor of proinflammatory cytokines. *CFTR* loss also impaired kidney reabsorption of amino acids and lactate and abolished renal glucose homeostasis. These and additional unexpected metabolic defects prior to disease manifestations reveal a fundamental role for *CFTR* in controlling multiorgan metabolism. Such discovery informs a basic understanding of CF, provides a foundation for future investigation, and has implications for developing therapies targeting only a single tissue.

## Abstract 2

Arteriovenous metabolomics in newborn cystic fibrosis pigs reveals an intrinsic role of the CFTR anion channel in controlling multiorgan metabolism.

## Graphical abstract

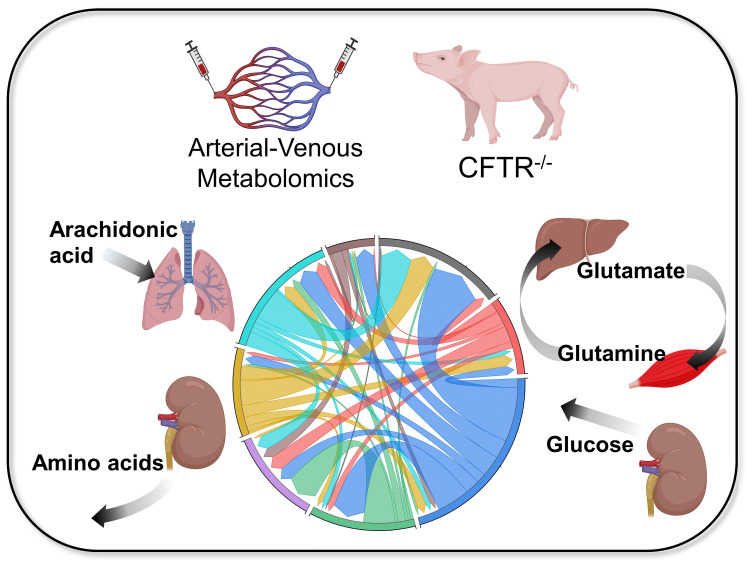

## Introduction

Cystic fibrosis (CF) is an autosomal recessive disease caused by loss-of-function mutations in the gene encoding the CF transmembrane conductance regulator (*CFTR*) anion channel (1–4). The clinical phenotype is influenced, in part, by specific *CFTR* mutations (5–9). However, the progression, severity, and outcomes of the disease are determined by multiple other factors that remain unidentified or poorly defined (8–10). Moreover, disease manifestations and metabolic defects have been described for nearly every organ, highlighting the importance of understanding the disease mechanisms across multiple organs.

In an attempt to better understand the broad diversity of metabolic manifestations, gain insight into the pathogenesis, and identify factors that predict disease severity, many studies have measured metabolites in CF (11–18). However, several factors have been limiting. First, people with CF have inflammation, infection, nutritional deficiencies, and other variable disease complications, and they receive multiple diverse treatments. These confounding factors prevent the identification of primary metabolic roles of *CFTR*. Second, most studies of metabolites have measured them in blood, urine, or bronchoalveolar lavage fluid, which leaves unresolved the contribution of specific organs. Third, studies in the commonly used laboratory mammal, mice, are limited because those with *CFTR* mutations do not recapitulate the clinical disease seen in humans (19). Fourth, studies are often limited by the measurement of only a few metabolites. Understanding how *CFTR* loss affects multiple organs has become increasingly important with the development of potential new treatments such as gene therapy that target a single organ or a cell type. However, to our knowledge, none of the studies has explored the direct function of *CFTR* in metabolic regulation across multiple organs comprehensively and systematically.

The most widely recognized CF metabolic abnormality is aberrant insulin secretion and the development of diabetes, usually after the late teens (20–23). However, whether these are due to pancreatic destruction or a primary defect due to loss of *CFTR* is debated (20–22). Given that *CFTR* is broadly expressed in many metabolically active organs and that abnormalities in various metabolites are observed in people with CF, we hypothesized that *CFTR* is a bona fide regulator of metabolism across multiple organs. To test this idea while circumventing the limitations of previous studies, we based our strategy on 3 approaches. First, we studied *CFTR*-deficient pigs (CF pigs). Pigs resemble humans in physiology, biochemistry, diet, circadian rhythm, and metabolic rates (24–29). The similarity between humans and pigs is further evidenced by the development of pigs as a source of organs for transplantation (30). Critically, unlike CF rodent models, CF pigs closely mirror human CF disease (31–34). Second, to elucidate metabolic changes that are inherently driven by *CFTR* deficiency and to avoid confounding effects of CF disease and treatment, we strategically focused on studying newborn CF pigs. Third, we used technically innovative arteriovenous (AV) metabolomics and in vivo stable isotope tracing with mass spectrometry to assess organ-specific uptake and release fluxes of hundreds of metabolites across 8 organs as well as dynamic cross-organ metabolite exchange.

Our results revealed that *CFTR* loss resulted in multiple unexpected metabolic flux changes across key organs including the lung, liver, muscle, and kidney. Critically, these disruptions were due to *CFTR* deficiency per se and occurred before disease symptoms. These findings have important implications for therapeutic development and will help guide future research.

## Results

### CFTR loss alters the circulating metabolome of newborn pigs.

**Figure 1:**
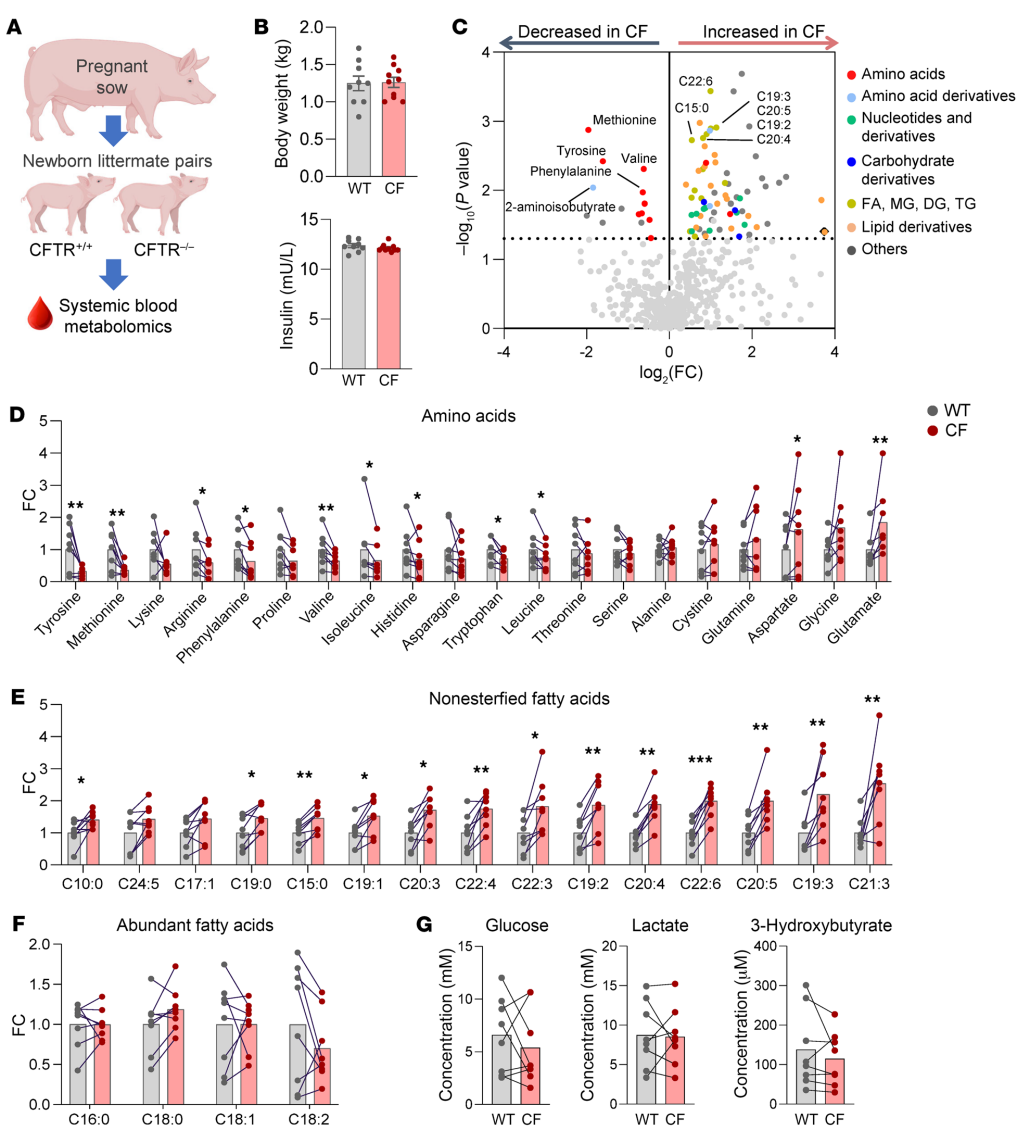
The circulating metabolome differs between WT and CF newborn pigs. (**A**) Experimental scheme for systemic blood metabolomics using LC-MS. (**B**) Body weights and fasting serum insulin levels (mU/L). *n* = 9 WT and *n* = 10 CF pigs. (**C**) Volcano plot indicating the difference of each circulating metabolite between WT and CF pigs (color coded to indicate various metabolite categories). Representative metabolites enriched in WT or CF are marked. Fatty acid (FA); monoglyceride (MG); diglyceride (DG); triglyceride (TG). (**D**–**G**) Comparison of amino acids (**D**), fatty acids (**E**), and other abundant circulating metabolite (**F** and **G**) levels in blood between 8 WT and CF littermate pairs. *P < 0.05, **P < 0.01, and ***P < 0.001 relative to WT, by 2-tailed, paired Student’s *t* test for metabolites meeting normality or Wilcoxon signed-rank test for metabolites that do not meet normality (see Supplemental Table 1). Bars show the mean. FC, fold change (CF/WT).

To investigate the intrinsic role of *CFTR* in metabolic regulation, we generated *CFTR^–/–^* (CF pigs) (31) and *CFTR^+/+^* pigs (WT pigs) of both sexes, including 8 pairs of littermates, and assessed the arterial blood metabolome of newborn piglets using liquid chromatography–mass spectrometry (LC-MS) (Figure 1A). Animals had not suckled for 3 hours before the study, and 2 litters were delivered by cesarean section and had not taken milk; there were no differences in the circulating metabolome of piglets that were delivered vaginally and those delivered by cesarean section.

Newborn WT and CF piglets had similar body weights and blood insulin levels (Figure 1B), in contrast to older CF pigs and people with CF who have reduced body weights due, at least in part, to abnormal nutrition (35). Despite the similar body weights and insulin levels, there were significant metabolome differences between genotypes, including decreased amino acids and increased nonesterified long-chain fatty acids (LCFAs) in CF piglets (Figure 1C and Supplemental Table 1 for FDR-corrected data; supplemental material available online with this article; https://doi.org/10.1172/JCI174500DS1). These findings prompted us to further examine amino acids and LCFAs systematically. Amino acids had mixed patterns, with some such as tyrosine showing a dramatic (~3-fold) decrease, whereas glutamate was (~2-fold) increased in CF piglets (Figure 1D). LCFAs showed more consistent patterns, with most of them substantially increased in CF piglets (Figure 1E) except for a few abundant fatty acids (Figure 1F). These amino acid and fatty acid abnormalities were specific because other major circulating metabolites including glucose, lactate, and ketones were relatively normal (Figure 1G).

### CFTR loss alters cross-organ metabolite exchange.

**Figure 2:**
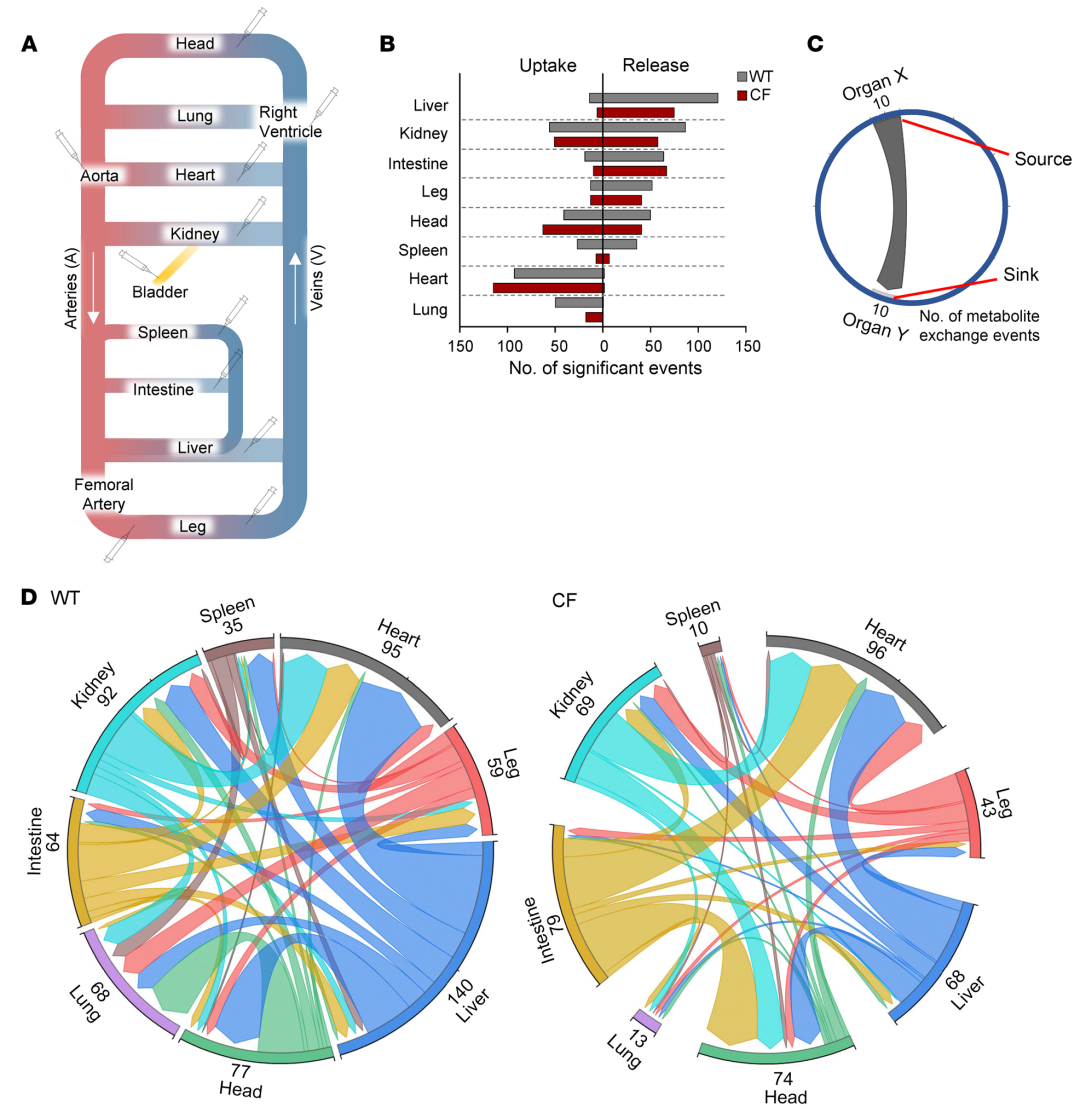
*CFTR* loss impairs metabolism in multiple organs and interorgan metabolite exchange. (**A**) Schematic of the circulatory system indicating blood and urine sampling sites. (**B**) For each organ in WT and CF pigs, metabolites showing significant release and uptake were identified using the 1-sample *t* test or Mann-Whitney *U* test (see Supplemental Table 2). (**C**) Schematic plotting the source and sink organs of circulating metabolites. The blunt end indicates source organ X, and the arrowed end indicates sink organ Y. Numbers indicate the number of metabolite exchange events that met statistical significance in both organs for each interorgan metabolite exchange. (**D**) Chord graphs indicating metabolite exchange between organs in WT and CF pigs. *n* = 9 WT and *n* = 10 CF pigs.

Our finding that *CFTR* loss altered the systemic blood metabolome in newborn piglets suggested an intrinsic role of *CFTR* in regulating metabolism. However, stationary blood metabolite levels do not provide information about the organs that drive such changes or the causal metabolic fluxes (e.g., metabolite production or consumption). Therefore, we used AV metabolomics to quantitatively determine metabolite uptake and release across multiple organs. We sampled arterial blood (aorta and femoral artery) and venous blood across 8 individual organs or body parts (lung, head, heart, spleen, kidney, liver, intestine, and the leg representing primarily skeletal muscle) (Figure 2A). We also collected fresh urine from the bladder to assess kidney function. All sampling was done within 30 minutes after the first blood draw. To augment the accuracy of the measurements, each biological sample was independently extracted and analyzed by LC-MS 3 times, and median AV values were used for data analysis. Then, to determine the net release and uptake of each metabolite by each organ, we calculated the log_2_ ratio of metabolite abundances in venous blood (*C_v_*) to those in arterial blood (*C_A_*). For the liver AV comparison, we used a weighted average of the hepatic artery (22%) and the portal vein (78%) relative to the hepatic vein (36). For the lung AV comparison, we used a ratio of arterial blood relative to the right ventricle.

These pan-organ, AV metabolite gradient measurements not only revealed dynamic metabolite exchange between organs in newborn WT pigs but also disclosed its altered patterns driven by *CFTR* loss (Supplemental Figure 1). Overall, the liver, kidney, and intestine released the greatest number of metabolites in both genotypes (Figure 2B and Supplemental Table 2), consistent with the roles of these organs in supplying circulating metabolites in adults (37, 38). By contrast, the heart, head, kidney, and lung absorbed the greatest number of metabolites. In many cases, CF reduced the number of metabolites released or absorbed by multiple organs such as the liver, spleen, and lung (Figure 2B).

As an example, compared with WT intestine and liver, CF intestine released less taurodeoxycholate (a bile acid) and the liver absorbed less (Table 1). This result suggests that *CFTR* is required for bile acid recycling and that its defect may contribute to serum bile acid abnormalities later in the disease course (39). In some cases, *CFTR* loss reversed metabolite trafficking. For example, although WT hearts released guanosine, CF hearts absorbed it. Inosine showed a similar trend. These purine nucleosides act as signaling molecules (40, 41) and their blood levels tended to decrease in CF (~50% and ~15%, Supplemental Table 1), suggesting altered communication between the heart and other organs by *CFTR* loss (Table 1 shows additional examples).

To further investigate the role of *CFTR* in regulating interorgan metabolite exchange, we inspected metabolites that showed statistically significant release by one organ and statistically significant uptake by another organ (Figure 2C and Supplemental Table 3). This analysis revealed that *CFTR* loss substantially disrupted metabolite exchange between organs (Figure 2D). Remarkably, in CF piglets, the number of metabolites exchanged between the liver and other organs decreased by half (from 140 to 68 metabolites), while the number of metabolites transferred to the lung from other organs decreased by 5-fold (from 68 to 13 metabolites) (Figure 2D).

### CFTR loss disrupts fatty acid uptake by the lung.

**Figure 3:**
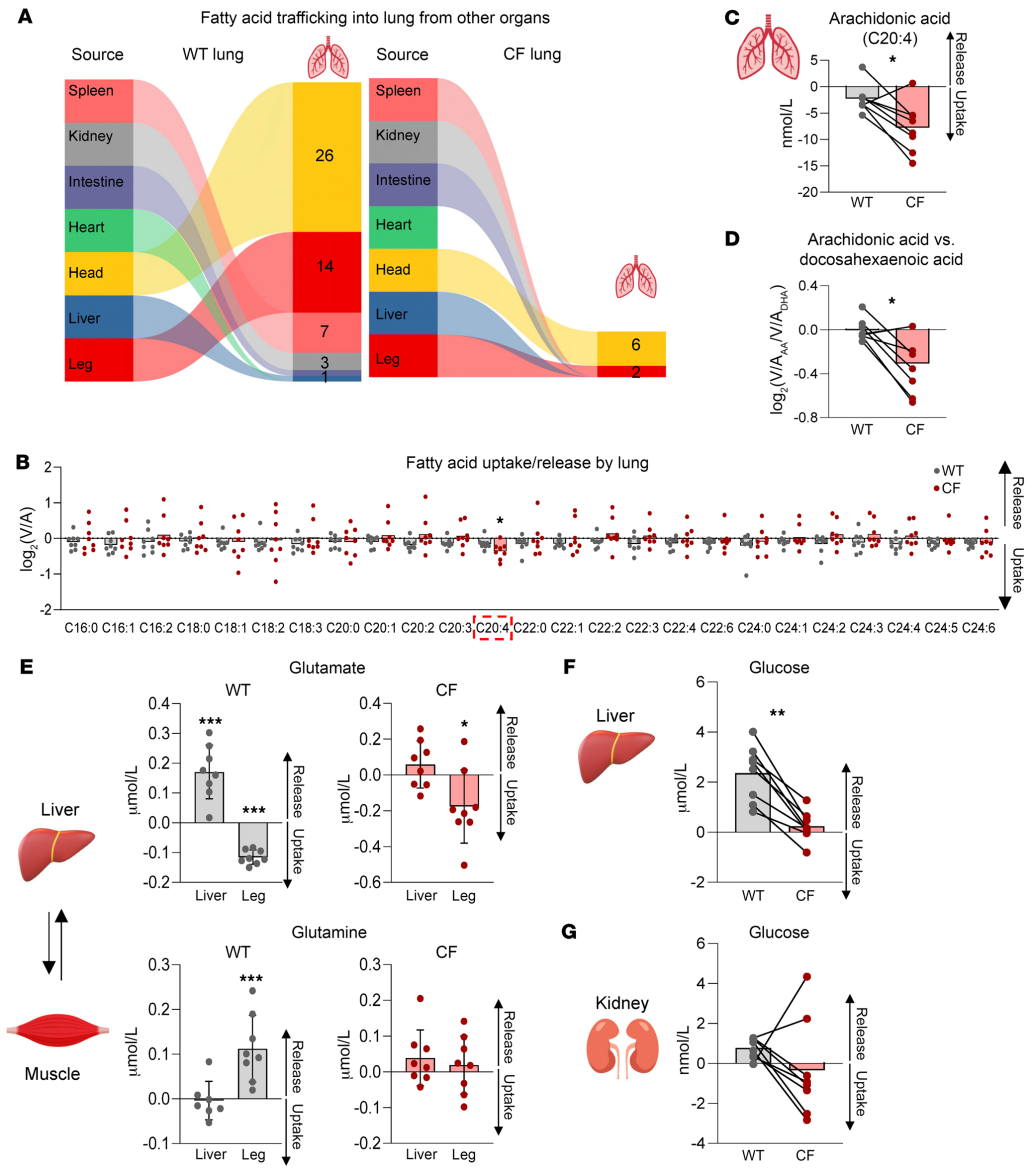
Quantitative analysis reveals altered metabolite trafficking in CF lungs, liver, and legs. (**A**) Graphs indicating fatty acid trafficking into lungs from other organs. (**B**) log_2_(V/A) ratios of LCFAs in WT and CF lungs. *n* = 8 WT and *n* = 8 CF littermates. Error bars show the mean. Note that arachidonic acid (C20:4) uniquely shows greater uptake by CF lung compared with WT lung. *P < 0.05, by paired, 2-tailed Student’s *t* test. (**C**) Arachidonic acid (C20:4) trafficking. Bars show the mean for 8 WT and 8 CF littermates. *P < 0.05, by paired, 2-tailed Student’s *t* test. (**D**) Calculated ratio of lung uptake of arachidonic acid versus docosahexaenoic acid. Bars show the mean. *P < 0.05, by Wilcoxon signed-rank test. *n* = 8 WT and *n* = 8 CF littermates. (**E**) Glutamate and glutamine trafficking between the liver and leg. Bars show the mean ± SD for 8 WT and 8 CF littermates, except glutamine WT liver (*n* = 7). *P < 0.05 and ***P < 0.001, by 1-sample *t* test or Mann-Whitney *U* test (see also Supplemental Table 2). (**F** and **G**) Hepatic and renal glucose trafficking. Bars show the mean. *n* = 8 WT and *n* = 8 CF littermates. **P < 0.01, by Wilcoxon signed-rank test.

This unexpected defect in lung uptake of circulating metabolites prompted us to further investigate the CF lung’s metabolic activities. Interestingly, most metabolites that showed blunted lung uptake turned out to be LCFAs (Figure 3A). Such defective LCFA uptake was unexpected, given the elevated levels of blood LCFAs in newborn CF piglets (Figure 1E). As developing lungs require LCFAs for surfactant production and other membrane remodeling processes, impaired lung LCFA uptake may contribute to later CF lung pathologies in adults.

Surprisingly, despite mostly defective lung LCFA uptake, arachidonic acid (C20:4) stood out as unique (Figure 3B), in that its uptake was enhanced by approximately 3-fold (from 2.5 to 7.5 nmol/L) by *CFTR* loss (Figure 3C). Arachidonic acid is an essential precursor of proinflammatory lipid cytokines (42). Indeed, an increased ratio of arachidonic acid to docosahexaenoic acid levels in adult CF lungs may contribute to a hyperinflammatory pulmonary response (43, 44). Our AV comparison revealed that this ratio was already increased in newborn CF piglets without inflammation. The data also suggested that such an increase was probably due to the increased lung “uptake” of arachidonic acid relative to docosahexaenoic acid (Figure 3D).

### CFTR loss disrupts liver and muscle metabolism.

We next switched our attention to other metabolically active organs and found that *CFTR* loss substantially disrupted the liver release of metabolites, especially amino acids and their derivatives (Supplemental Table 3). One notable example was glutamate. In WT piglets, the liver released a significant amount of glutamate (~0.17 μmol/L), which was taken up by the leg (Figure 3E). WT piglet leg, in turn, released glutamine (~0.1 μmol/L), which was approximately 60% (at a molar mass) of the absorbed glutamate. This liver-muscle exchange of glutamate and glutamine is critical for the removal of ammonia in muscle via the urea cycle in liver (45), which promotes muscle energy metabolism including amino acid catabolism (38, 46). Our data indicate that this process occurred even in newborn pigs, but the loss of *CFTR* disrupted it. These findings may explain an earlier observation that patients with CF show impaired muscle energy metabolism (15).

Another unexpected observation was substantially less glucose release by the liver in the absence of functional *CFTR* (Figure 3F). Kidneys, a secondary gluconeogenic organ, also showed less and inconsistent glucose release (Figure 3G). After lung disease and the complications of lung transplantation, liver disease has been the major cause of mortality in people with CF (47). Histological analyses have variably shown focal biliary cirrhosis, hepatic steatosis, and/or noncirrhotic portal hypertension (48). Although cholangiocytes and, to a lesser extent, hepatocytes express *CFTR*, the pathophysiological basis by which loss of *CFTR* causes liver disease is poorly understood (49). Our findings of metabolic abnormalities in the newborn liver, prior to secondary manifestations from CF disease and its treatment, can provide new opportunities and directions to explore the responsible mechanisms.

### CFTR loss disrupts homeostatic metabolite release by kidneys.

**Figure 4:**
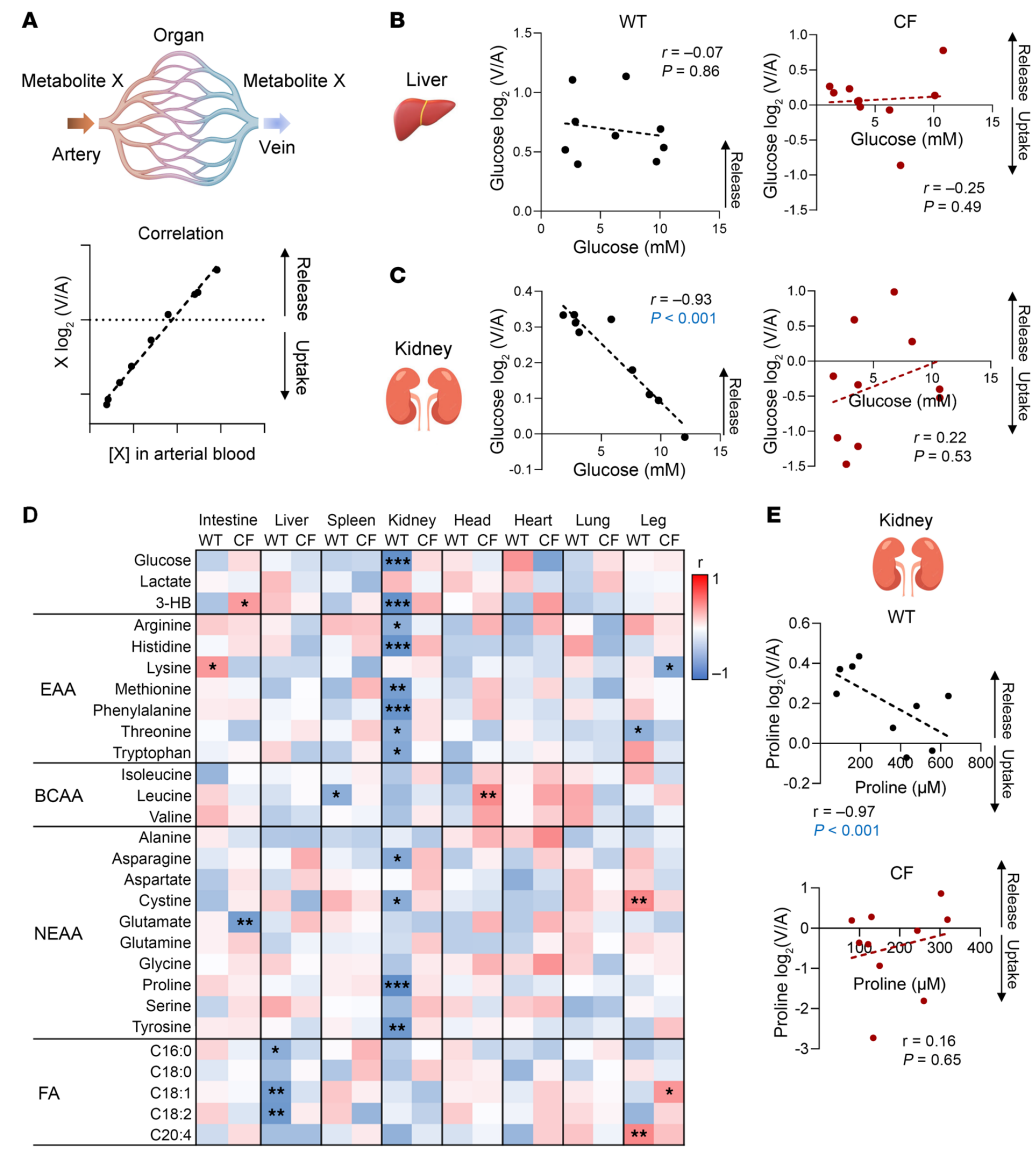
Loss of *CFTR* disrupts renal glucose and amino acid homeostasis. (**A**) Schematic of correlation analysis of circulating metabolites between arterial concentration, [X], and tissue release/uptake activity [log_2_(V/A)]. For correlation analysis in the liver, the weighted average of portal and arterial blood concentration was used. (**B** and **C**) Correlations between blood glucose concentration and hepatic or renal release in WT and CF pigs. *P* values were calculated by Spearman’s correlation test. *n* = 9 WT and *n* = 10 CF pigs. (**D**) Heatmap showing correlations for 28 abundant metabolites across 8 organs. EAA, essential amino acids; BCAA, branched-chain amino acids; NEAA, nonessential amino acids. *P < 0.05, **P < 0.01, and ***P < 0.001, by Spearman’s correlation test. *n* = 9 WT and *n* = 10 CF pigs. (**E**) Correlations between arterial blood concentrations and kidney trafficking of proline in WT and CF pigs. *P* values were calculated by Spearman’s correlation test. *n* = 9 WT and *n* = 10 CF pigs.

Metabolite levels are tightly maintained within physiological ranges by homeostatic mechanisms including hormonal regulation and concentration-dependent oxidation (50). To gain insight into how *CFTR* loss disrupts systemic metabolite levels, we performed an unbiased correlation-based analysis between each metabolite’s blood concentration and uptake/release by each organ (Figure 4A and Supplemental Table 4). In the liver, neither WT nor CF showed a significant relationship between blood glucose concentration and release (Figure 4B); perhaps this is a feature of newborn pigs (51). In contrast, in WT kidney, we found a strong (*P* < 0.001) inverse relationship; that is, the lower the blood glucose concentration, the greater the renal glucose release (Figure 4C), suggesting homeostatic regulation. Strikingly, *CFTR* loss disrupted this relationship.

Motivated by the glucose result, we expanded our analysis to the other 27 metabolites for which we measured absolute concentration values. For WT organs, we found 20 statistically significant relationships (Figure 4D). Surprisingly, 60% (12 cases) involved the kidney, all 12 were inverse correlations, 10 were for amino acids, and the other 2 were for glucose and 3-hydroxybutyrate. In all 12 cases, loss of *CFTR* disrupted the correlation (Figure 4E shows proline as an example).

### CFTR loss disrupts renal amino acid reabsorption.

**Figure 5:**
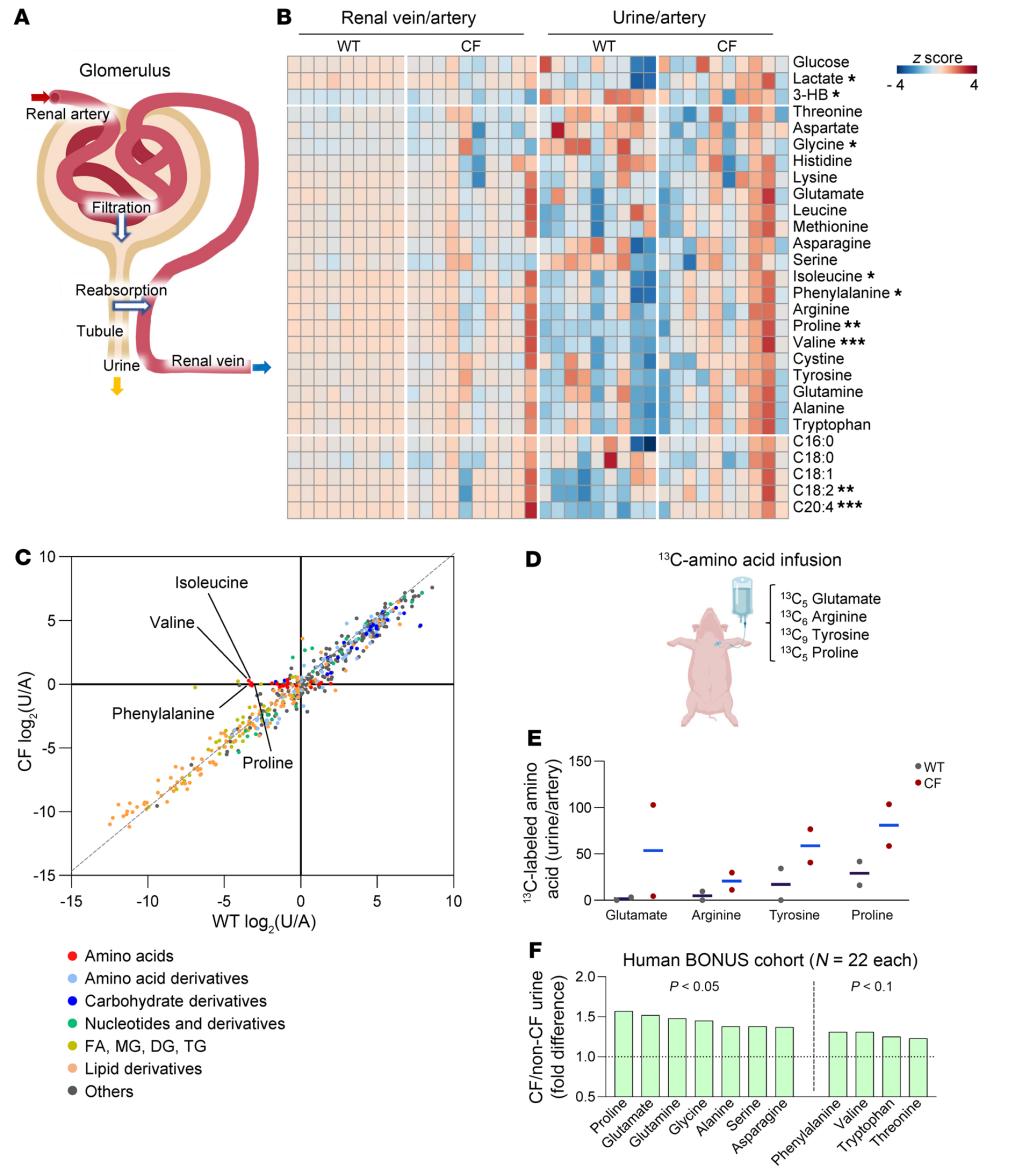
CF kidney exhibits defective amino acid reabsorption. (**A**) Schematic of renal filtration and reabsorption. Most metabolites are first filtered, and selected metabolites are then actively reabsorbed back into the systemic circulation. (**B**) Heatmap showing 28 metabolite abundance ratios of the renal vein relative to the artery and urine relative to the artery. Blue color in urine/artery indicates reabsorption, and red color indicates loss into urine. *P < 0.05, **P < 0.01, and ***P < 0.001 of CF urine/artery relative to WT, by 2-tailed Student’s *t* test or Mann-Whitney *U* test (see Supplemental Table 2). Urine data were normalized to urine creatinine levels. (**C**) Correlation between WT urine/artery and CF urine/artery for each circulating metabolite (color coded by categories). Examples of amino acids that are poorly reabsorbed in CF are labeled. (**D**) Schematic of stable isotope tracing in pigs. Pigs were intravenously infused with four ^13^C-labeled amino acid tracers. (**E**) ^13^C-labeled amino acid abundance in WT and CF urine samples. The ion counts of ^13^C-labeled amino acids in urine (normalized to urine creatinine) were normalized to the labeled amino acids in arterial blood. Data are individual points with the mean shown by a blue line. *n* = 2 WT and *n* = 2 CF littermates. (**F**) Fold difference in urine amino acids, normalized by urine osmolality, from 12-month-old children with CF (*n* = 22) relative to non-CF controls (*n* = 22). *P* values by Welch’s 2-sample *t* test. Data were adapted from BONUS (12).

These findings led us to further investigate the role of *CFTR* in kidney function. The renal glomerulus filters circulating metabolites, then proximal tubule epithelia selectively reabsorb crucial metabolites including glucose and amino acids and allow others to escape into the urine (52) (Figure 5A). Renal venous versus arterial blood comparisons did not reveal consistent abnormalities across CF kidneys, probably because variations in metabolite abundance were too small to detect statistically significant differences (Figure 5B). However, urine-to-arterial metabolite ratios revealed less efficient reabsorption (i.e., increased loss to urine) by CF kidney of multiple metabolites, including lactate and several amino acids (Figure 5B). Global comparison of urine versus blood metabolomes between WT and CF piglets further indicated that amino acids were inefficiently reabsorbed in the absence of *CFTR* (Figure 5C).

The glomerular filtration rate (GFR) has been reported to be normal or decreased in CF (53, 54). However, reports of a decrease in GFR are associated with use of nephrotoxic agents for pulmonary infection, use of immunosuppressive drugs after lung transplantation, and CF-related diabetes (55), conditions not present in newborn pigs. Consistent with a normal GFR, except for some amino acids, most of the approximately 400 metabolites showed similar abundances in WT and CF urine (Figure 5C). As an additional test, we assessed blood metabolite indicators that have a strong correlation with the estimated GFR (eGFR) (Supplemental Figure 2) (56). However, we found no difference by genotype, further indicating that the GFR is probably not impaired in newborn CF piglets.

To measure renal amino acid reabsorption more directly, we infused WT and CF piglets intravenously with 4 stable isotope–labeled ^13^C–amino acid tracers, selected on the basis of the sizes, charges, and polarity that determine transporter specificities (Figure 5D). After an initial bolus and continuous infusion with minimal perturbation of circulating concentrations (Supplemental Figure 3), we collected fresh urine and measured the leakage of the intravenously infused tracers into the urine. CF pigs showed an increase in all 4 traced amino acids in the urine (Figure 5E).

We also investigated metabolites in urine that were collected from 12-month-old children with or without CF in the Baby Observational and Nutrition Study (BONUS) (12). We examined the data set from that study and found significantly higher levels of several amino acids in CF urine (Figure 5F). Together, we conclude that loss of *CFTR* impairs renal amino acid reabsorption.

### Inhibition of CFTR reduces amino acid transport by proximal tubule epithelia.

**Figure 6:**
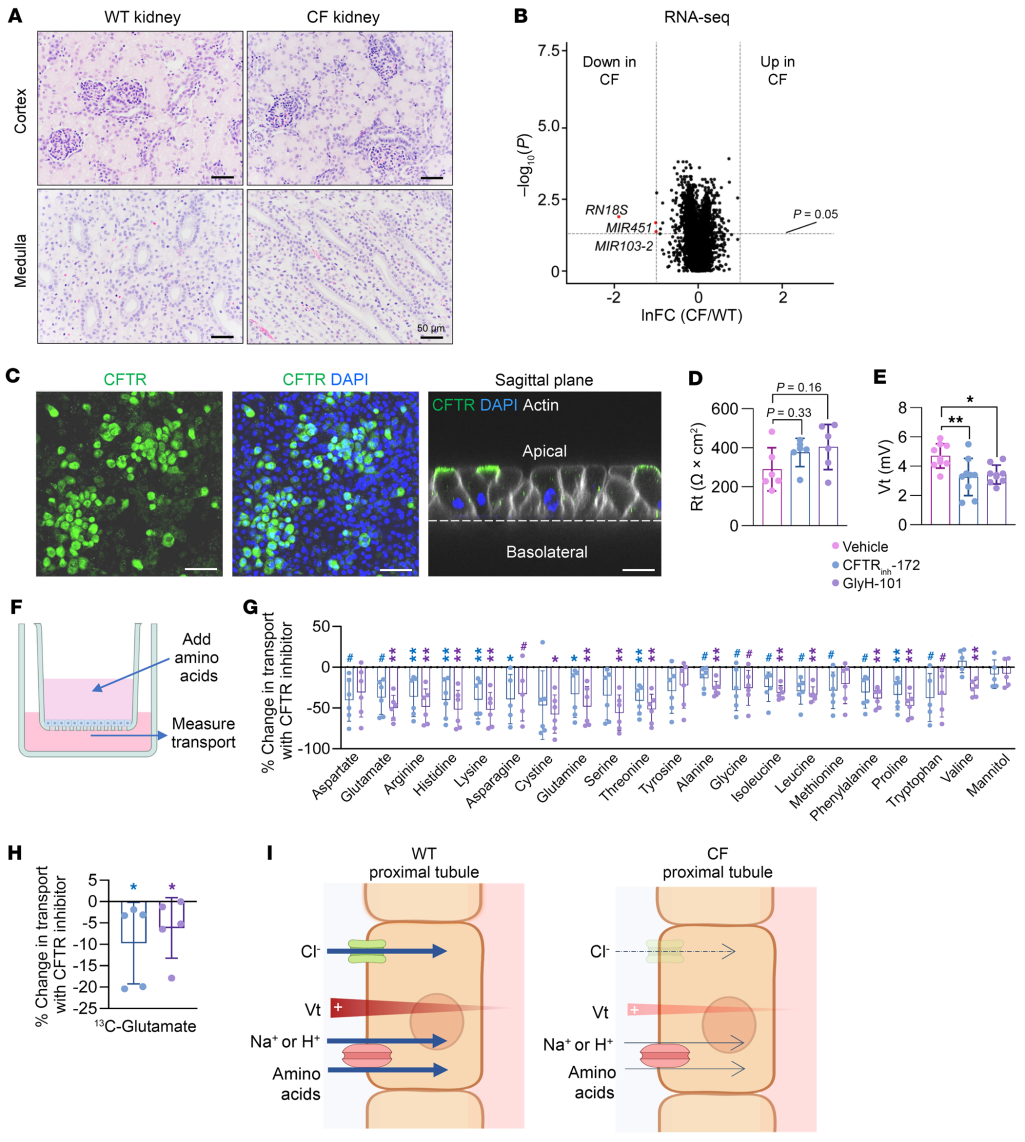
Inhibition of CFTR in cultured human renal proximal tubule epithelia impairs amino acid absorption. (**A**) H&E staining of newborn WT and CF pig kidneys. Scale bars: 50 μm. (**B**) Volcano plot showing differentially expressed genes in WT and CF pig kidneys. lnFC, fold change (CF/WT) in kidney tissues at the natural log scale. Dashed lines indicate the cutoffs: genes that were lower in CF with *P* < 0.05, by Mann-Whitney *U* test are highlighted in red. *n* = 12 WT and 11 CF pigs. Down, downregulated; Up, upregulated. (**C**) Immunofluorescence staining for CFTR (green) and actin (white) in DAPI-labeled human renal proximal tubule epithelial cells. Scale bars: 20 μm (left 2 panels) and 100 μm (right-most panel). (**D** and **E**) Rt and Vt in cells after addition of vehicle control or 2 CFTR inhibitors (CFTR_inh_-172 or GlyH-101). *n* = 6 per group for Rt and *n* = 9 per group for voltage. Bars show the mean ± SD. *P < 0.05 and **P < 0.01, by 1-way ANOVA. (**F**) Schematic of an assay to measure transepithelial transport of amino acids. (**G** and **H**) Percentage change in transepithelial transport of amino acids by CFTR inhibitors at 60 minutes after amino acid addition. Mannitol was used as a negative control. ^#^*P* < 0.1, *P < 0.05, and **P < 0.01 for color-coded inhibitors relative to vehicle, by Mann-Whitney *U* test. Bars show the mean ± SD. *n* = 6 per group, except *n* = 5 for valine, asparagine, tryptophan, mannitol, and ^13^C-glutamate. (**I**) Proposed model for impaired amino acid reabsorption by the proximal tubule. Cl^–^ absorption through CFTR anion channels contributes to the lumen positive voltage, and loss of CFTR decreases voltage. The positive voltage enhances Na^+^- and H^+^-coupled amino acid reabsorption.

We next sought to investigate the mechanisms of how *CFTR* loss disrupts renal amino acid reabsorption. Proximal tubule epithelia use a variety of membrane transporters to reabsorb amino acids. To first test whether transporter expression is affected by *CFTR* loss, we quantitatively examined gene expression of 56 known amino acid transporters of WT and CF kidney cortex but found no significant differences (Supplemental Figure 4A). We next examined kidney histopathology but found no obvious difference between WT and CF (Figure 6A). Global mRNA transcripts measured by RNA-Seq also showed little genotype-dependent differences (Figure 6B). We further investigated protein expression and localization of select amino acid transporters. Western blotting and immunofluorescence staining showed no effect by *CFTR* deficiency on either protein levels or localization of the glutamate transporter (SLC1A1) or the neutral amino acid transporter (SLC38A4) (Supplemental Figure 4, B–E). However, we cannot exclude the possibility of changes in other protein levels. Collectively, these results suggest that impaired amino acid reabsorption is not simply attributable to altered renal structure, development, or transporter expression.

To further investigate the biochemical mechanisms underlying impaired renal amino acid reabsorption by *CFTR* loss, we used human renal proximal tubule epithelial cells cultured on permeable membrane supports (57). After the cells had formed epithelia, immunofluorescence imaging confirmed CFTR expression at the apical membrane (Figure 6C). Blocking CFTR function with either of 2 pharmacological inhibitors, CFTR_inh_-172 or GlyH-101 (58, 59), tended to increase transepithelial electrical resistance (Rt) while significantly decreasing the lumen-positive transepithelial voltage (Vt) (Figure 6, D and E), indicating that CFTR ion channels influence the electrophysical properties of proximal tubule epithelia. To measure amino acid transport in this condition, we then added 20 amino acids and mannitol (as a negative control) to the apical solution and measured their appearance in the basolateral solution (Figure 6F). We observed that inhibiting CFTR significantly decreased the absorptive flux of almost all amino acids but not mannitol (Figure 6G).

To determine whether inhibiting CFTR decreases glutamate flux from the apical to the basolateral surface versus inhibiting the release of glutamate produced internally by the cells (60), we added ^13^C-glutamate to the apical surface and measured its basolateral appearance. We found that inhibiting CFTR reduced ^13^C-glutamate transport, indicating inhibition of transepithelial glutamate absorption (Figure 6H).

Individual amino acid transporters can carry a variety of different amino acids, and many couple amino acid flux to Na^+^ or H^+^ flux. In kidneys, such Na^+^ and H^+^ electrochemical gradients across membranes power amino acid reabsorption (61). Thus, we propose a model in which the loss of CFTR ion channels in renal proximal tubule impairs amino acid reabsorption via disruption of electrochemical gradients (Figure 6I). The loss of amino acids may have contributed to the impaired renal glucose homeostasis that we observed in CF piglets (Figure 4C) due to abnormal gluconeogenesis using amino acids as substrates (62–64).

## Discussion

CF has not typically been considered a metabolic disease. By measuring AV gradients of numerous metabolites across multiple organs and interorgan metabolite exchange in a relevant disease model prior to the disease’s secondary manifestations, this study revealed the intrinsic role of *CFTR* in regulating organ metabolism. These findings challenge the traditional view that metabolic abnormalities are only or primarily the consequences of CF progression.

Our results indicate marked abnormalities in lung LCFA uptake by *CFTR* loss. Previous studies reported altered LCFA levels in people with CF; linoleic acid (C18:2), eicosatrienoic acid (C20:3), docosahexaenoic acid (C22:6), and arachidonic acid (C20:4) are the LCFAs most often cited (43, 65–70). However, dietary fat malabsorption due to pancreatic failure and altered intestinal function cloud interpretation in human studies. Intestinal abnormalities in CF mouse models and potential epigenetic changes in cells cultured from people with CF also limit the interpretation of such studies. Our data indicate that, even before body weight loss, insulin dysregulation, or development of disease manifestations, loss of *CFTR* per se changes systemic LCFA metabolism. Among many LCFAs, arachidonic acid (C20:4) uniquely shows enhanced lung uptake by *CFTR* loss. The arachidonic acid abnormality reported in CF has largely been regarded as a secondary result of lung inflammation and infection. Our data, however, suggest that *CFTR* loss per se in the lung augmented arachidonic acid uptake prior to inflammation or infection.

Arachidonic acid is an essential precursor of proinflammatory lipid cytokines such as prostaglandins and leukotrienes (42). Previous research showed that loss of *CFTR*-mediated HCO_3_^–^ and Cl^–^ transport across airway epithelia impairs respiratory host defenses precipitating bacterial lung infections (1, 33, 34, 71, 72). Thus, when the airways do become infected, an intrinsic abnormality in arachidonic acid metabolism may predispose CF lungs to profuse inflammation, a hallmark of the disease. Accordingly, the antiinflammatory drug ibuprofen, which inhibits arachidonic acid conversion to prostaglandins, has shown benefit for CF lung disease (73).

We had not anticipated the important role of *CFTR* in regulating renal metabolism. Comparison of the urine versus blood metabolomes indicated that CF kidneys exhibit inefficient reabsorption of many amino acids, a conclusion supported by our ^13^C-amino acid tracer infusion study. It was striking to find a similar loss of amino acids in the urine of infants with CF (12). The renal proximal tubule where amino acid reabsorption occurs is the site of *CFTR* expression in the kidneys. Influenced by mechanistic renal proximal tubule research published nearly 50 years ago (61), we found that lack of a CFTR anion conductance may change the driving force for Na^+^- and H^+^-coupled amino acid absorption in the proximal tubule.

Surprisingly, loss of *CFTR* also disrupted renal glucose homeostasis; low systemic glucose concentrations failed to provoke renal glucose release. Renal gluconeogenesis also occurs in the proximal tubule and relies predominantly on lactate and amino acids. Thus, the impaired reabsorption of these gluconeogenic substrates by *CFTR* loss may contribute to dysregulation of renal gluconeogenesis. It is well known that people with CF who are not diabetic or are not using medicines to lower glucose levels can develop spontaneous episodes of hypoglycemia (74, 75). Although historically this has been attributed to reactive hyperglycemia and dysregulated insulin secretion, the pathophysiology is poorly understood, and faulty renal gluconeogenesis may contribute, at least in part, to this abnormality.

This study has advantages and limitations. Using newborn pigs has the major advantage that it can reveal the poorly understood intrinsic roles of CFTR ion channels in regulating organ metabolism (our major goal). In this way, we eliminated confounding interpretations due to secondary consequences of the disease, treatments, and compensatory changes, although we cannot exclude the potential effect of *CFTR* loss on prenatal organ development. In addition, we did not investigate how *CFTR* loss changes multiple organ metabolism during disease progression, which will be equally important. Another critical advantage of using newborn pigs is the paired comparison of littermates, thereby reducing genetic variation; unlike laboratory mice but like humans, the pigs are not an inbred strain. Use of littermates also minimizes environmental variations; such a paired comparison is less valid in adults because environmental influences often go unappreciated. We also strategically focused on fasting conditions to avoid secondary effects of different nutrient absorption between WT and CF piglets, but it will be important to elucidate the role of *CFTR* in regulating postprandial metabolism. A limitation is that AV metabolomics do not identify the cell types responsible for metabolic flux abnormalities; novel technologies including imaging mass spectrometry will be required to provide such granular information (76). Another limitation is that anesthesia is indispensable to collect venous blood draining from deeply buried newborn organs. To mitigate the effect of anesthesia on metabolism, we maintained body temperature, blood pressure, and blood volume, i.e., routine clinical procedures (77). Importantly, data from human AV studies of patients under anesthesia have been remarkably similar to data obtained from individuals without anesthesia (78–80), suggesting that these procedures can successfully minimize anesthesia effects.

This study may also encourage investigators to assess the metabolic effects of other channels or gene products, even in diseases in which metabolic abnormalities are not anticipated or are complicated by secondary effects. Our results provide a compelling demonstration that combining state-of-the-art technology (unbiased and comprehensive AV metabolomics) and a relevant model can reveal surprising new insights into cross-organ metabolic interactions in a genetic disease. CFTR is an ion channel, not a metabolic enzyme. However, because ion channels are such efficient machines, even a small amount of activity can change membrane voltages and/or ion concentrations; we provide a mechanistic example in renal amino acid absorption.

The recent introduction of medicines that target mutant *CFTR* has brought profound health benefits to people with CF (81–84). These individuals have experienced dramatic improvements in their lung disease, the major cause of morbidity and mortality. Other aspects of their health have also benefited, including readily quantified variables such as increased body weight, as well as many variables that are more difficult to objectively quantify. Nevertheless, approximately 10%–15% of people with CF have unresponsive mutations or adverse reactions to current *CFTR*-modulating medications. As a result, there are major efforts to develop alternative treatments that target the pulmonary airways, where disease is the major cause of mortality (85–88). Our results indicate that treatments targeting only airway epithelia will leave unaffected CF metabolic abnormalities across multiple other organs. What the long-term consequences may be for disease progression and severity are uncertain. However, the results of this research also lay the groundwork for further investigation and understanding of the metabolic consequences of *CFTR* loss.

## Methods

### Sex as a biological variant.

We used 5 male and 5 female CF piglets, and 4 male and 5 female WT piglets. In 6 of the 9 littermate pairs, we were able to match the sex of the pair. For isotope infusion experiments, we used 1 male CF piglet and 1 female CF piglet and 1 male WT piglet and 1 female WT piglet.

### Animals.

*CFTR^+/–^* pigs were mated to produce *CFTR^–/–^* piglets. Piglets were obtained from Exemplar Genetics and studied within 12 hours of birth. For AV metabolomics experiments, 10 *CFTR^–/–^* piglets (referred to as CF pigs) and 8 *CFTR^+/+^* and 1 *CFTR^+/–^* piglets (referred to as WT pigs) from 9 litters were used (25). For paired experiments, 8 *CFTR^–/–^* piglets and 8 of their *CFTR^+/+^* littermates were studied.

### Animal experiments.

Newborn piglets were sedated/anesthetized with inhaled isoflurane at 2%–4%. Oxygen saturation (SpO_2_) and heart rate were monitored continuously, and 0.9% saline was infused at 2–6 mL/kg/h intravenously to maintain intravenous fluids. Standard surgical procedures were used to access blood vessels in the abdomen and chest. The subcutaneous tissues and muscle layers were carefully dissected. Hemostasis was achieved with electrocautery. Blood was drawn from each vessel using a 27 or 30 gauge needle connected to a 1 mL syringe. Residual urine was removed from the bladder at the beginning of the surgery, and fresh urine was collected at the end using a 27 gauge needle. After the completion of blood sampling, the animal was euthanized. Blood samples were placed on ice and centrifuged at 1,500*g* for 15 minutes to obtain serum. Samples were stored at –80°C until LC-MS analysis. For insulin quantitation, an Ultra Sensitive Porcine Insulin ELISA Kit (10–1200–01, Mercodia) was used.

### Isotope tracer infusion.

Saline (0.9% NaCl) containing 15 mM [U-^13^C]-glutamate, 12 mM [U-^13^C]-proline, 5 mM [U-^13^C]-arginine, and 4.5 mM [U-^13^C]-tyrosine was infused intravenously via the ear vein. Blood was collected from the femoral or carotid artery at 5- to 10-minute intervals during the infusion. An initial bolus infusion was performed with 6 mL/kg/h for 20 minutes before surgery, and the infusion rate was decreased to 2.4 mL/kg/h during surgery for 50 minutes to achieve steady-state labeling.

### Metabolomics by LC-MS.

Arterial blood (10 μL), venous blood, and urine were extracted with 300 μL ice-cold acetonitrile/methanol/water (40:40:20) solution. Following vortexing and centrifugation at 16,000*g* for 10 minutes at 4°C, 70 μL supernatant was loaded into MS vials. Metabolites were analyzed by quadrupole-orbitrap mass spectrometer (Thermo Fisher Scientific) coupled to hydrophilic interaction chromatography (HILIC) via electrospray ionization. LC separation was on an Xbridge BEH amide column (2.1 mm × 150 mm, 2.5 μm particle size, 130 Å pore size; Waters) at 25°C using a gradient of solvent A (5% acetonitrile in water with 20 mM ammonium acetate and 20 mM ammonium hydroxide) and solvent B (100% acetonitrile). The flow rate was 150 μL/min. The LC gradient was: 0 minutes, 90% B; 2 minutes, 90% B; 3 minutes, 75% B; 7 minutes, 75% B; 8 minutes, 70% B; 9 minutes, 70% B; 10 minutes, 50% B; 12 minutes, 50% B; 13 minutes, 25% B; 14 minutes, 20% B; 15 minutes, 20% B; 16 minutes, 0% B; 20.5 minutes, 0% B; 21 minutes, 90% B; and 25 minutes, 90% B. The autosampler temperature was set at 4°C, and the injection volume of the sample was 3 μL. MS data were acquired in negative and positive ion modes with a full-scan mode from *m/z* 70–830 with 140,000 resolution. Data were analyzed using the Compound Discoverer (Thermo Fisher Scientific) and MAVEN software. Metabolites were confirmed by authentic standards based on retention time, precursor ion, and tandem mass spectrum generated in-house and the Human Metabolome Database. For systemic blood metabolomics in Figure 1, arterial serum samples from all pigs were analyzed as a single batch to avoid the LC-MS batch effect. For AV metabolomics in Figures 2–5, arterial and venous serum from each pig was analyzed as a single batch for accurate AV ratio calculations. To correct technical variables such as metabolite extraction yield and LC-MS injections, an internal standard (^15^N-valine) was spiked into the extraction solvent. In addition, to identify any systematic errors during LC-MS runs and to calculate the coefficient of variation (CV), pooled serum samples were generated for quality control (QC) and repetitively injected among approximately 20 samples. Metabolites showing a CV greater than 50 were excluded from the analysis.

### Renal proximal tubule cell culture.

Human primary renal proximal tubule epithelial cells (89) were purchased from the American Type Culture Collection (ATCC) (PCS-400-010). Cells were plated on a human collagen IV–coated 10 cm dish and cultured in Renal Epithelial Cell Basal Media supplemented with Renal Epithelial Cell Growth Kit components (both from ATCC), as per ATCC’s protocols. At 80% density, cells were trypsinized and seeded on human collagen IV–coated 0.33 cm^2^ Transwell permeable membranes (3470, Corning) at 200,000 cells/cm^2^. Previous studies utilized an air-liquid interface (ALI) culture technique to promote the organization of renal epithelial cells (90, 91). Those studies noted that, in contrast to the traditional submerged conditions, the ALI culture approach markedly improved epithelial cell polarity, tubular cell formation, and maturation. Therefore, we adopted this approach. Two days after seeding, apical media were removed, and cells were maintained in an ALI culture for 3 weeks while being fed basolaterally every other day with the complete growth media (91). Rt and Vt were measured with the Epithelial Volt/Ohm Meter (World Precision Instruments).

### Western blot analysis.

Snap-frozen kidney cortex tissue from newborn piglets was homogenized in ice-cold RIPA buffer (150 mM NaCl, 1% IGEPAL CA-630, 0.5% sodium deoxycholate, 0.1% SDS, 50 mM Tris-HCl, pH 8.0) supplemented with Protease Inhibitor Cocktail (Roche) and 2 mM sodium vanadate, 20 mM sodium fluoride, and 20 mM β-glycerophosphate. Protein lysates were quantified using the Pierce BCA protein assay kit (Thermo Fisher Scientific) and transferred onto Immobilon-FL PVDF membranes (MilliporeSigma). Membranes were incubated with primary antibodies overnight at 4°C in 5% BSA in Tris-buffered saline Tween 20 (TBST) (50 mM Tris–HCl, 150 mM NaCl, 0.05% Tween-20, pH 8.0). After primary antibody binding, membranes were washed 3 times with TBST and probed with anti-rabbit (IRDye 800CW, LI-COR Biosciences, 1:10,000) and anti-mouse (IRDye 680RD, LI-COR Biosciences, 1:10,000) secondary antibodies in TBST for 1 hour at room temperature. After washing 3 times with TBST, proteins were visualized using the Odyssey Infrared Imaging System (LI-COR Biosciences). The following primary antibodies were used: rabbit anti-SLC1A1 (12686-1-AP, Proteintech, 1:1,000); rabbit anti-SLC38A4 (20857-1-AP, Proteintech, 1:1,000); and mouse anti–β-actin (A2228, clone AC-74, MilliporeSigma, 1:5,000).

### Immunofluorescence.

For staining on tissue, kidney cortex from newborn piglets was fixed in 10% buffered formalin and then paraffin embedded. Sections were deparaffinized through successive incubations in xylene and decreasing concentrations of ethanol. Antigens were then retrieved by boiling the tissue sections in 10 mM citrate buffer, pH 6.0. For staining on Transwell cultures, cells were washed once with PBS and fixed in 4% paraformaldehyde for 1 hour at room temperature. After fixation and antigen retrieval (for tissue sections), cells were permeabilized with 0.2% Triton X-100 (Thermo Fisher Scientific) in PBS for 20 minutes and then blocked in Super-Block (Thermo Fisher Scientific) with 5% normal goat serum for 1 hour. Cells were incubated with primary antibodies overnight at 4°C, then with anti-mouse secondary antibody conjugated to Alexa Fluor 488 (Molecular Probes/Invitrogen, 1:1,000) or anti-rabbit secondary antibody conjugated to Alexa Fluor 568 (Molecular Probes/Invitrogen, 1:1,000), and with phalloidin conjugated to Alexa Fluor 633 (Molecular Probes/Invitrogen, 1:100) for 1 hour at room temperature. Tissue sections and Transwell membranes were mounted with Vectashield plus DAPI (Vector Laboratories) and imaged with an Olympus FluoView FV3000 confocal microscope. The following primary antibodies were used: mouse anti-CFTR (clone 769, University of North Carolina – Chapel Hill and the Cystic Fibrosis Foundation Therapeutics, 1:200); rabbit anti- SLC1A1 (12686-1-AP, Proteintech, 1:1,000); and rabbit anti-SLC38A4 (20857-1-AP, Proteintech, 1:1,000).

### In vitro amino acid transport assay.

Krebs-HCO_3_ buffer solution (118.9 mM NaCl, 25 mM NaHCO_3_, 2.4 mM K_2_HPO_4_, 0.6 mM KH_2_PO_4_, 1.2 mM MgCl_2_, 1.2 mM CaCl_2_, 5 mM dextrose, at 37°C adjusted to pH 7.4 in the presence of 5% CO_2_) containing 20 amino acids or mannitol (1 mM each) was added apically to epithelia cultured on Transwells, and the basolateral solution was collected after 60 minutes. To maintain the same osmolarity in apical and basolateral solutions, mannitol was added to the basolateral solution except when mannitol was solely added to the apical solution as a negative control experiment.

### RNA-Seq.

Kidney cortex was dissected, and RNA was extracted using a RNeasy Lipid Tissue Kit (QIAGEN) according to the manufacturer’s directions. After digestion of genomic DNA with DNase I (QIAGEN), RNA concentration was quantified using fluorometry (Qubit 2.0 fluorometer; Life Technologies, Thermo Fisher Scientific), and quality was assessed using an Agilent BioAnalyzer 2100 (Agilent Technologies). Ribosomal RNA depletion and sample library preparation were done using the Illumina TruSeq Stranded Total RNA with RiboZero, followed by sequencing on a NovaSeq 6000 (Illumina). The quality of raw sequencing data was assessed using FastQC. All samples passed the QC analysis. Raw sequencing reads were pseudoaligned to the pig genome reference Sscrofa11.1, and the abundance of transcripts was quantified using the Kallisto, version 0.48.0, Python program (92). Transcript counts were imported into R using the Sleuth R package (93). Counts of different transcripts for the same gene were aggregated. Genes with differential expression between non-CF and CF samples were identified using the Mann-Whitney *U* test.

### Histology.

Fixed tissues were dehydrated through a progressive series of ethanol and xylene baths, paraffin embedded into blocks, and then sectioned (~4 μm) and stained with H&E stain. Tissues were examined by a pathologist using the post-examination method of masking (94). High-resolution digital images were collected using a BX53 microscope, a DP73 digital camera, and CellSens Dimension software (Olympus).

### Statistics.

Metabolite ion counts were log_2_ transformed to enhance the normal distribution of data. If the normal distribution was observed in both groups (*P* < 0.05, by Shapiro-Wilk normality test), a 2-tailed, paired Student’s *t* test was used to calculate *P* values between WT and their CF littermates. If the normal distribution was not observed in one of the groups, Wilcoxon signed-rank test was used. Likewise, a 1-sample *t* test was used to calculate the *P* values of organ-specific metabolite trafficking events if the normal distribution was observed. If not, a 1-sample Mann–Whitney *U* test was used. For FDR correction, the Benjamini-Hochberg procedure was used to calculate *q* values for each series of sample types.

For chord graph analysis in Figure 2, the pair-wise relationship between organs was met if statistics in the source organ [log_2_(V/A) > 0, *P* < 0.05] and sink organ [log_2_(V/A) < 0, *P* < 0.05] matched. For graph analysis of lung fatty acid trafficking in Figure 3A, the pair-wise relationship between organs was met if statistics in the source organ [log_2_(V/A) > 0, *P* < 0.1] and sink organ [log_2_(V/A) < 0, *P* < 0.1] matched. Outliers were determined by boundaries ± 2.5 SDs from the mean. For correlation analysis, *P* values were calculated by Spearman’s correlation test. A *P* value of less than 0.05 was considered significant, and the data are present mean ± SD.

### Study approval.

The animal studies followed protocols approved by the University of Iowa IACUC (approval no. 0081121).

### Data availability.

RNA-Seq data are available in the Gene Expression Omnibus (GEO) database (GEO GSE217122). Values for all data points in graphs are reported in the Supporting Data Values file.

## Author contributions

CJ and MJW conceived the project and supervised the study. BRK and DVDH performed animal experiments and cell culture experiments. HB and SJ performed the sample processing and LC-MS analysis. JL contributed to data analysis. WY performed and analyzed the mRNA experiments. SHP, TK, and DKM helped with sample preparation and data analysis. BMH, NDG, LSP, and DKM assisted with animal experiments. VLS contributed to the model development. HB, BRK, CJ, and MJW wrote the manuscript. All authors read the manuscript and provided comments. HB, BRK, and SJ contributed equally to the work and share co–first authorship. The authorship order was assigned on the basis of the degree of contribution.

## Supplementary Material

Supplemental data

Unedited blot and gel images

Supplemental table 1

Supplemental table 2

Supplemental table 3

Supplemental table 4

Supporting data values

## Figures and Tables

**Table 1:**
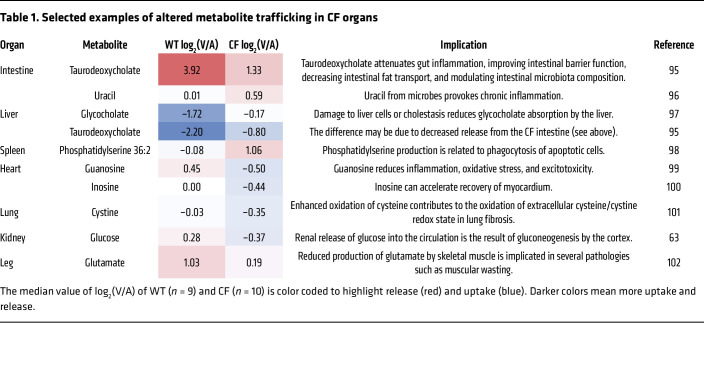
Selected examples of altered metabolite trafficking in CF organs

## Version 1 05/14/2024

In-Press Preview

## Version 2 07/01/2024

Electronic publication
