# Supplementary material for: Arteriovenous metabolomics in pigs reveals CFTR regulation of metabolism in multiple organs
Source: J Clin Invest. 2024 May 14;134(13):e174500. doi: 10.1172/JCI174500 (PMC11213515; doi:10.1172/JCI174500)
Supplement: Supplemental data [file jci-134-174500-s108.pdf]

Supplemental Figure 1

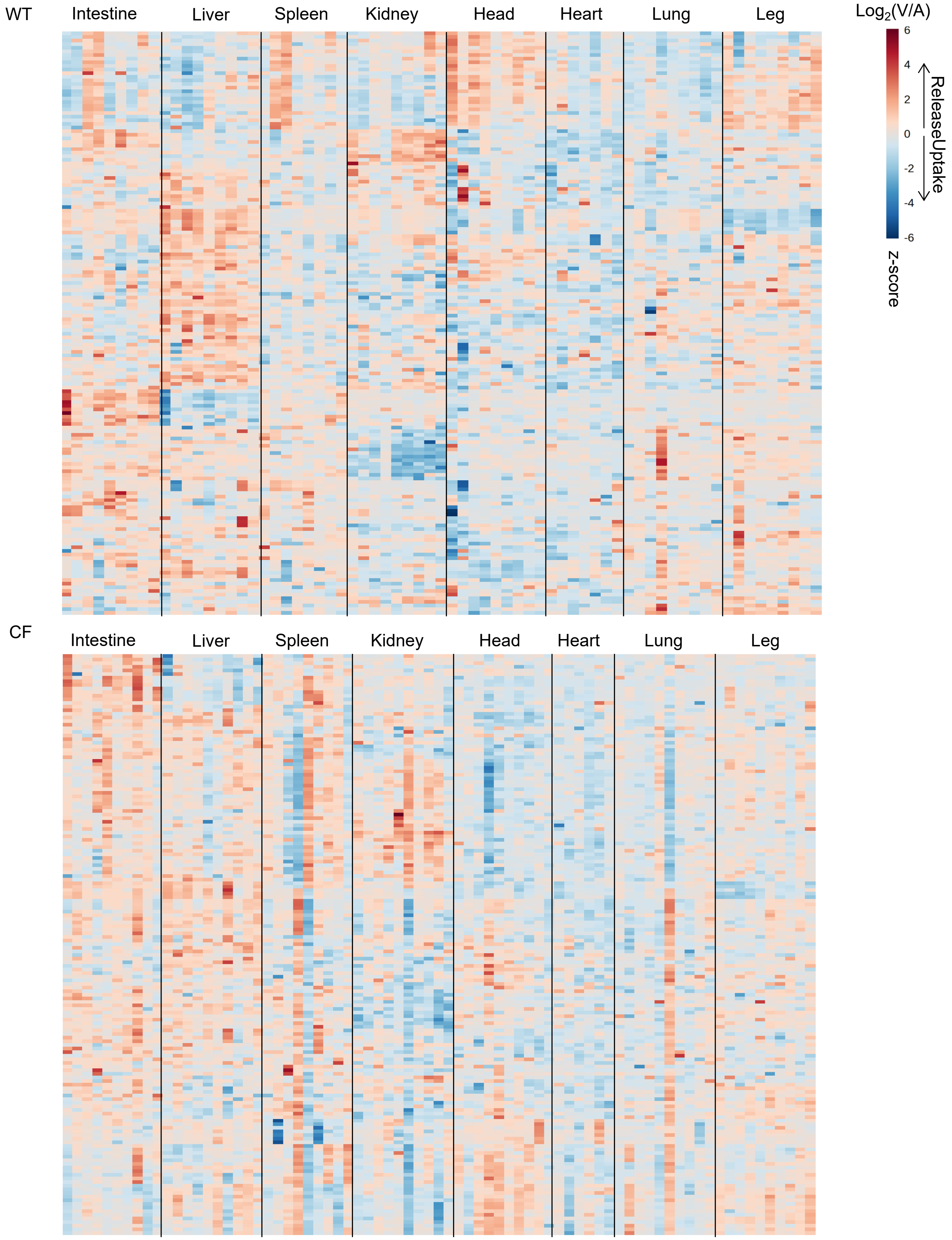

Supplemental Figure 1. Heatmap of 161 circulating metabolites that showed significant trafficking related to Figure 2. Heatmap of 161 circulating metabolites that showed significant trafficking at least in one organ, determined by FDR<0.05 by ANOVA. Each data point indicates a median of three technical replicates. Head and legs likely reflect metabolic activities of the brain and skeletal muscle respectively. N=9 WT and 10 CF pigs except for WT heart (N=7) and spleen (N=8), and CF heart (N=6) and spleen (N=9).

Supplemental Figure 2

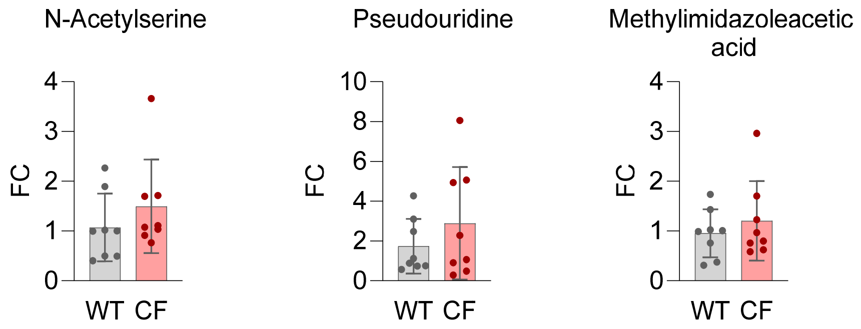

Supplemental Figure 2. No change in eGFR indicators between WT and CF new born pigs, related to Figure 5
Comparison of blood metabolite indicators that have a strong correlation with eGFR between 8 WT and CF littermates. Bars show mean±s.d

Supplemental Figure 3

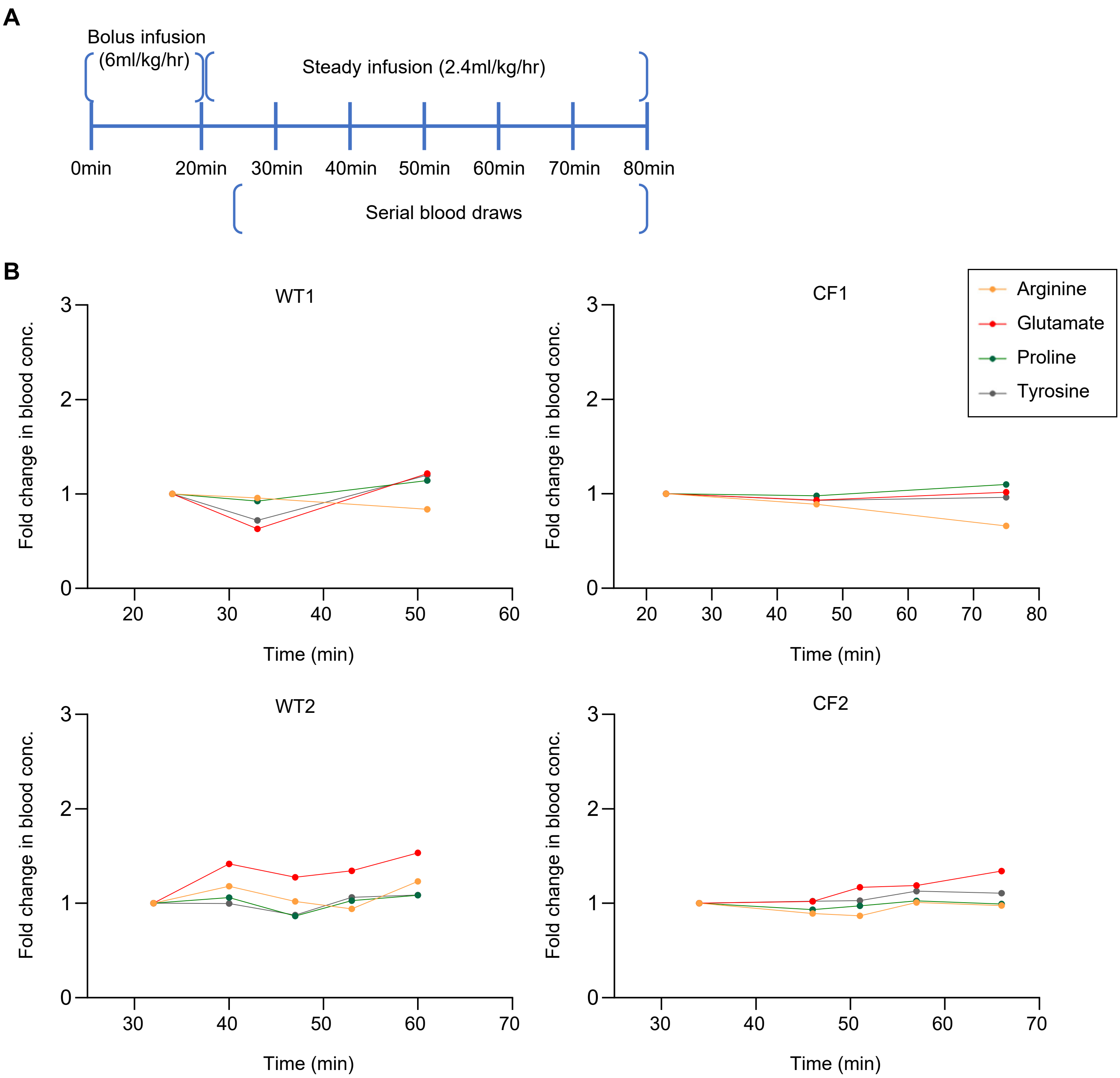

Supplemental Figure 3. Stable isotope tracer infusions in pigs, related to Figure 5
(A) Schematic of ^{13}C-amino acid isotope tracer infusions. After a 20 min bolus infusion, pigs were infused at a steady rate, and arterial blood was drawn at serial intervals. **(B)** Time-course fold change in blood concentrations of the infused amino acids (sum of ^{13}C-labeled and unlabeled forms) relative to the first draw for each pig.

Supplemental Figure 4

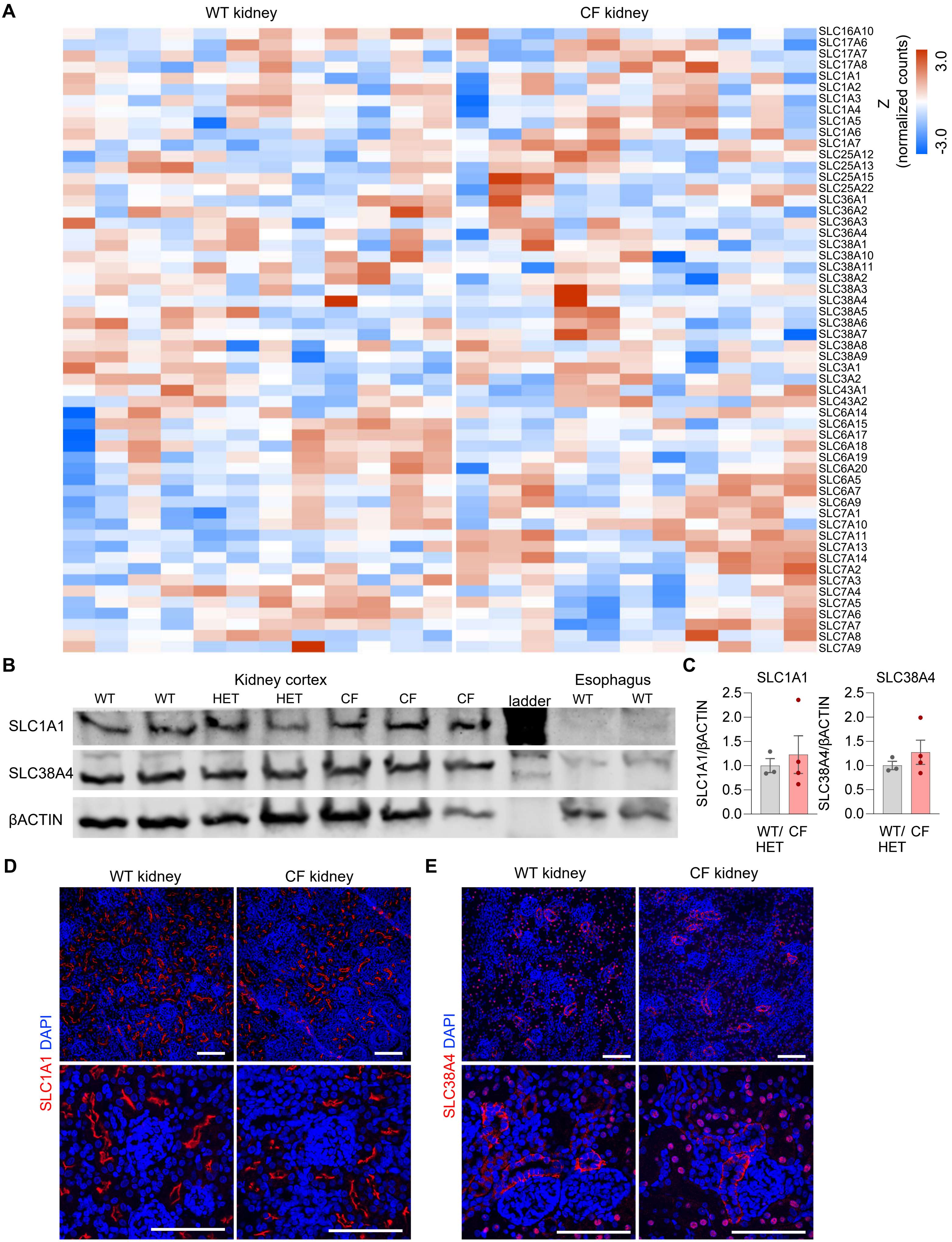

Supplemental Figure 4. CF kidney shows similar mRNA and protein expression, and localization of amino acid transporters, related to Figure 6

(A) Heatmap showing normalized counts of amino acid transporter mRNA in WT and CF kidneys. N=12 WT and 11 CF pigs. **(B-C)** Immunoblot analysis of SLC1A1 and SLC38A4 on WT/HET and CF kidney cortex tissues. Esophagus was used as a negative control. Quantitative data are presented on the right **(C)**. Bars show mean±s.d. N=4 for WT/HET and 3 for CF. **(D-E)** Immunofluorescence staining of SLC1A1 **(D)** and SLC38A4 **(E)** on WT and CF kidney proximal epithelial tissues. Scale bars, 100 µm.
