# Supplementary figures and images for: Arteriovenous metabolomics in pigs reveals CFTR regulation of metabolism in multiple organs
Source: J Clin Invest. 2024 May 14;134(13):e174500. doi: 10.1172/JCI174500 (PMC11213515; doi:10.1172/JCI174500)

Full unedited gel for Supplemental Figure 4B

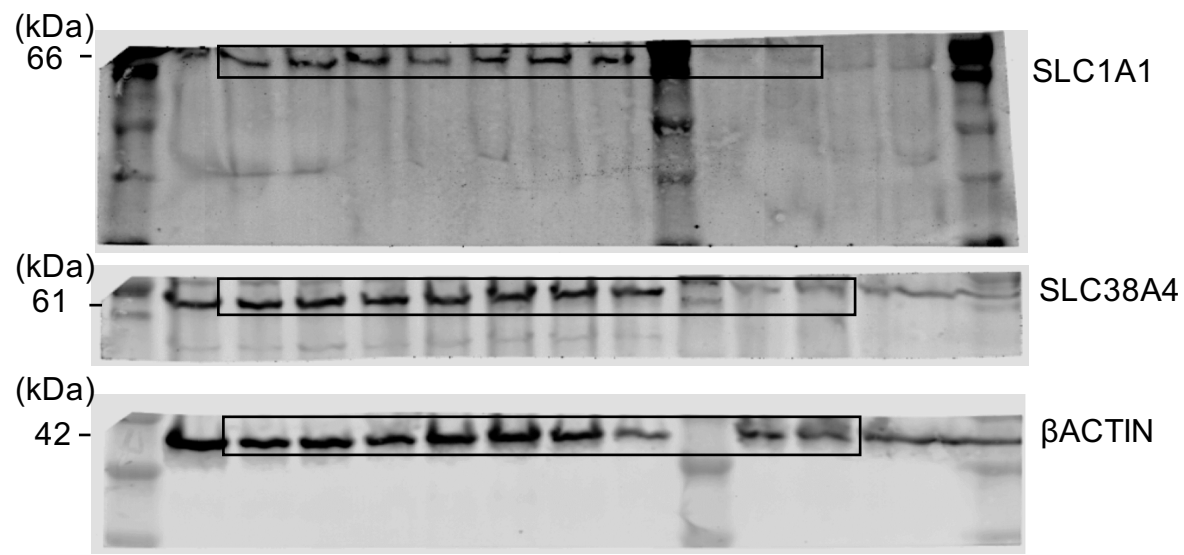

Supplement: Unedited blot and gel images [file jci-134-174500-s109.pdf]
